# Immunogenicity of a polyvalent HIV-1 candidate vaccine based on fourteen wild type gp120 proteins in golden hamsters

**DOI:** 10.1186/1471-2172-7-25

**Published:** 2006-10-31

**Authors:** Ali Azizi, David E Anderson, Masoud Ghorbani, Katrina Gee, Francisco Diaz-Mitoma

**Affiliations:** 1 Infectious Disease and Vaccine Research Centre, Research Institute Children's Hospital of Eastern Ontario, 401 Smyth Road, Ottawa, ON, K1H 8L1, Canada; 2 Variation Biotechnologies Inc., 22 de Varennes, Suite 210, Gatineau, Quebec, J8T 8R1, Canada; 3 Center for Neurologic Diseases, Brigham and Women's Hospital, Harvard Medical School, 77 Avenue Louis Pasteur, Boston, MA, 02115, USA

## Abstract

**Background:**

One of the major obstacles in the design of an effective vaccine against HIV-1 is the hypervariability of the HIV-1 envelope glycoprotein. Most HIV-1 vaccine candidates have utilized envelope glycoprotein from a single virus isolate, but to date, none of them elicited broadly reactive humoral immunity. Herein, we hypothesised that a cocktail of HIV-1 gp120 proteins containing multiple epitopes may increase the breadth of immune responses against HIV-1. We compared and evaluated the immunogenicity of HIV-1 vaccines containing either gp120 protein alone or in combinations of four or fourteen gp120s from different primary HIV-1 isolates in immunized hamsters.

**Results:**

We amplified and characterized 14 different gp120s from primary subtype B isolates with both syncytium and non-syncytium inducing properties, and expressed the proteins in Chinese Hamster Ovary (CHO) cell lines. Purified proteins were used either alone or in combinations of four or fourteen different gp120s to vaccinate golden hamsters. The polyvalent vaccine showed higher antibody titers to HIV-1 subtype B isolates MN and SF162 compared to the groups that received one or four gp120 proteins. However, the polyvalent vaccine was not able to show higher neutralizing antibody responses against HIV-1 primary isolates. Interestingly, the polyvalent vaccine group had the highest proliferative immune responses and showed a substantial proportion of cross-subtype CD4 reactivity to HIV-1 subtypes B, C, and A/E

**Conclusion:**

Although the polyvalent approach achieved only a modest increase in the breadth of humoral and cellular immunity, the qualitative change in the vaccine (14 vs. 1 gp120) resulted in a quantitative improvement in vaccine-induced immunity.

## Background

HIV-1 gp120 is a major target for neutralizing antibodies (Nabs) and for this reason it is an important HIV immunogen to include in vaccine formulations [1-3]. However, the diversity of gp120 has proven to be a significant challenge to HIV-1 vaccine development. The structure of gp120 contains variable loops (V1-V5) which likely hide critical conserved epitope sites favoured by the Nabs. Furthermore, the crystallography structure of gp120 indicates that the protein is covered by carbohydrates which facilitates viral escape from Nabs [4,5]. Genetic variability in HIV-gp120 between groups M, N and O also affect the induction of Nabs [6,7]. These factors complicate the design of an effective candidate vaccine against HIV.

Previous vaccine studies focus on single HIV immunogens and although some of these studies show an increase in CD4/CD8+T cell immune responses, the immunogens used were not able to induce potent Nabs that mediate sterilizing immunity [8,9]. The question remains: "can a single immunogen induce a broad immune response against a diverse virus like HIV". To address this, several studies have been performed. A single and double recombinant HIV-1 gp120 protein has been used as a candidate immunogen in a phase III clinical vaccine trial. However, this vaccination was not effective to protect against HIV infection [10-12]. This lack of vaccine efficacy may be due to HIV diversity. While some single immunogens neutralize a few T-cell line adapted (TCLA) HIV-1 strains, none of the animal model or clinical studies demonstrated a broadly cross-reactive immunity against HIV-1 primary isolates [13]. Some studies demonstrated neutralizing antibody responses against HIV-1 primary isolates, however no measure of cross-reactivity was obtained as the strains of HIV virus used in the Nab assay, contained the same HIV-1 gp120 as that used for vaccination.

HIV-1 subtype B is widely distributed throughout the world and is the most common subtype in North America and Europe [14,15]. Herein, we hypothesised that immunization with several (fourteen) different wild type HIV-1 gp120 subtype B proteins would increase the breadth of specific antiviral immune responses. Fourteen wild type HIV-1 gp120 subtypes B were amplified, cloned and the recombinant gp120 proteins were expressed in mammalian cell lines. Golden hamsters were immunized with equivalent amounts of 1 vs. 4 vs. 14 distinct gp120 proteins and humoral (antibody binding and neutralization) and cellular (T helper cells) responses to HIV-1 subtypes B, C and A/E were analyzed. Although this polyvalent approach achieved only a modest increase in the breadth of humoral and cellular immunity; the qualitative change in the vaccine (14 vs. 1 gp120, same amount of total antigen) resulted in a quantitative improvement in vaccine-induced immunity.

## Results

### Characterization and expression of HIV-1 gp120s

**Figure 1:**
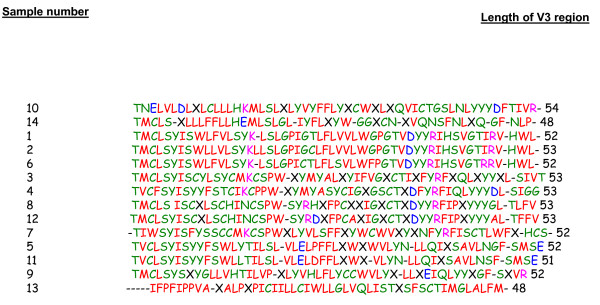
Alignment of fourteen amino acid sequences from V3 region of HIV-1 gp120 genes. Nucleotide sequences of V3 region of HIV-1 gp120 genes were translated and aligned by using ClustalW multiple sequence alignment program. Highly variation was seen in the number and position of HIV-1 V3 regions.

**Figure 2:**
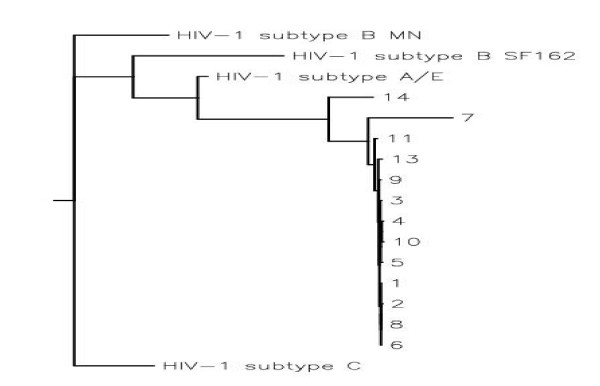
Phylogenetic relationships of HIV-1 gp120 sequences with subtypes B (MN, SF162), C (96ZM651), and A/E (93TH975). The algorithm is applied to a longitudinal data set of the C2-V5 regions of HIV-1 gp120s using Neighbor Tree-Maker in the FASTA format.

**Figure 3:**
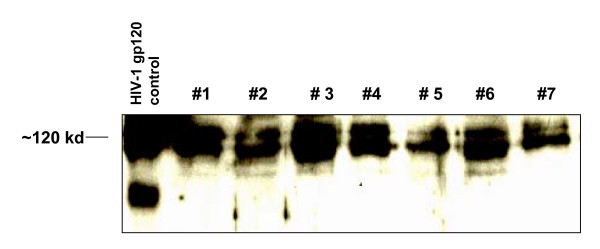
Western blot analysis of recombinant HIV-1 gp120 proteins. Lane 1: control gp120 protein expressed in a baculovirus expression system obtained from NIH. The lanes 2–8 are the first seven gp120 proteins (#1–7) purified from transfected CHO cells. The blot was probed with polyclonal gp120 antibody.

Total RNA was purified from syncytium and non-syncytium inducing co-cultures of 14 HIV-1 patients (Table 1). Amplification products corresponding to the full length of gp120 (1.6 kb) containing constant and hypervariable regions were generated by RT-PCR. The genes were completely sequenced and identified as subtype B. The V3 amino acid sequence of the amplified gp120s was compared with V3 subtypes B, C and A/E, point mutations as well as insertion and deletion mutations were detected (Fig 1). In addition, the phylogenetic relations between the 14 different gp120 sequences and HIV subtypes B, C and A/E were revealed and genetic diversity between clones was identified (Fig 2). Due to the limited number of nucleotides in the V3 region, the algorithm is applied to a longitudinal data set of the C2-V5 regions of HIV-1 gp120s using Neighbor Tree-Maker in the FASTA format. By this approach, epidemiological linkage was established between the HIV-1 gp120 clones and HIV-1 subtypes B, C and A/E. Most of the HIV-1 gp120 sequences were clustered from the HIV-1 subtype B isolate MN. This isolate was epidemiologically linked to the subtype A/E isolate 93TH975. The results revealed no particular homology between gp120 clones and HIV-1 subtype C isolate 96ZM651. Each gp120 variant was cloned into the pEF6-myc-His vector for the expression and purification of recombinant gp120 proteins *in vitro*. Stable CHO cell lines transfected with the gp120 constructs selected for using blasticidine resistance and recombinant gp120 proteins were purified by metal affinity chromatography. The expression of all recombinant HIV-1 gp120 proteins has been determined by western blotting (Fig 3).

**Table 1:** Viruses were isolated from the above HIV-1 seropositive patients.

**Sample**	**Sex**	**Virus isolate**	**Antiviral therapy**
1	M	R5	Y
2	M	R5	Y
3	M	R5	Y
4	F	R5	Y
5	M	R5	Y
6	M	R5	Y
7	M	R5	Y
8	M	R5	Y
9	M	R5	Y
10	M	R5	Y
11	M	X4	Y
12	M	X4	Y
13	M	X4	Y
14	F	X4	Y

### Immunization schedule

Four groups of hamsters were immunized with different vaccine formulations as indicated in Table 2. In the first group (A), hamsters were immunized with MPL adjuvant alone. In the second group (B), hamsters were immunized with 100 μg of the recombinant gp120 protein purified from patient number 001 plus adjuvant. Hamsters in the third group (C) were immunized with a mixture of proteins purified from patients #001 to #004 (25 μg of each recombinant gp120 protein) and adjuvant. The polyvalent group (D) immunized with a cocktail of all 14 proteins (~7 μg of each recombinant gp120 protein). Hamsters were immunized subcutaneously (S.C.) on weeks 0, 4, and 8. Animals were rested for 8 weeks and then boosted at week 16 with the same combination of gp120 proteins. Antibody titers were determined two weeks after each immunization.

**Table 2:** Immunization strategies: Groups of four hamsters were immunized four times S.C. in three different sites with one month interval between each immunization.

**Group A**	MPL adjuvant
**Group B**	HIV-gp120 protein #1 + MPL adjuvant
**Group C**	HIV-gp120 protein #1–4 + MPL adjuvant
**Group D**	HIV-gp120 protein #1–14 + MPL adjuvant

A total of 100 μg recombinant protein in 0.2 mg of monophosphoryl lipid A was injected per animal.

### The polyvalent candidate vaccine induces higher antibody titers against HIV-1 subtype B than other subtypes

**Figure 4:**
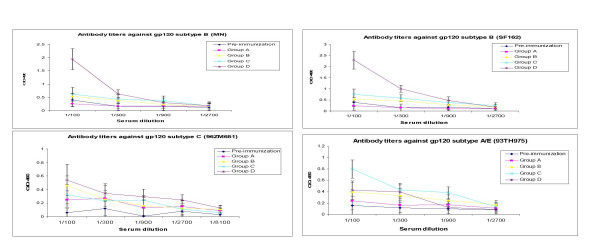
Antibody titers were determined in hamsters (n = 4) two weeks after the last immunization. The 96-well plates were coated with standard HIV-1 gp120 proteins (subtypes B, C and A/E). Hamster sera were serially diluted in wells and absorbance was read at 490 nm with an ELISA plate reader. Results are shown as mean concentration. ± S.E. is shown for each group.

In order to measure antibody titers against various HIV-1 gp120 proteins, plasma from immunized hamsters was collected two weeks after each immunization and analyzed by ELISA. The plates were coated with HIV-1 gp120 subtypes B (MN and SF162), C (96ZM651), and A/E (93TH975) overnight at 4°C followed by incubation with serially diluted plasma and developed as described in the Materials and Method section. The first two immunizations elicited a low level of antibody response to gp120 proteins (data not shown). However, after the third immunization, antibodies were detectable in all groups immunized with gp120. As expected the control group did not produce an antibody response. The highest level of antibody titer was observed in hamsters that were immunized with 14 recombinant gp120 proteins. In contrast, the control group (adjuvant alone) did not develop detectable levels of specific antibody responses. Eight weeks after the third immunization, animals were vaccinated again with respective recombinant gp120 proteins. Results similar to the third immunization were observed against gp120 subtypes between the groups. The polyvalent vaccine group induced higher levels of anti-gp120 titers to MN and SF162 (subtypes B) than the subtypes C and A/E (Fig 4). These results demonstrate that the polyvalent vaccine induces a higher antibody response to subtype B than the single gp120 candidate vaccine.

### Measurement of neutralizing antibody responses in immunized hamsters

**Figure 5:**
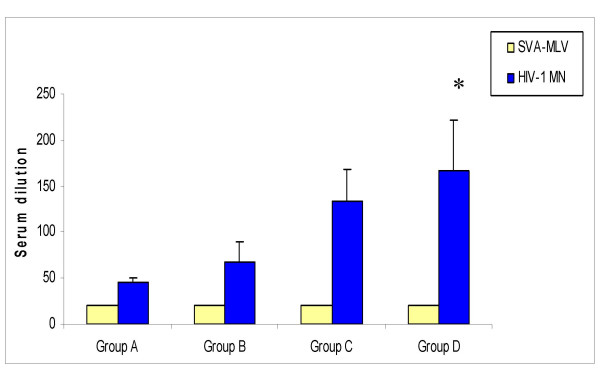
Neutralizing antibody responses in immunized groups. Values are the serum dilution at which relative luminescence units (RLU) were reduced 50% compared to virus control wells. The neutralization antibody responses were compared with SVA-MLV (Murine retrovirus Env-pseudovirus) as a background control for non-specific activity. Symbol * indicates significant difference when compared with groups A and B (*p *< 0.05).

Two weeks after the fourth immunization, sera were collected and Nabs to HIV-1 strains were tested. Unlike many research studies, to detect the real breadth and strength of candidate vaccines, we used different HIV-1 strains, to assay for Nabs, from those, which were used in immunization. Since some background was observed in the pre-bleed samples, the Nab responses were compared with SVA-MLV (murine retrovirus Env-pseudovirus) as a background control for non-specific activity. The group of hamsters that received all 14 gp120 proteins showed higher Nabs titres to the HIV-1 MN strain than the single gp120 (*p *< 0.05), however, similar Nabs titres were observed in the group immunized with a combination of four gp120 proteins (Fig 5). Sera from immunized hamsters were further tested against other primary HIV-1 isolates but none of the sera were able to show significant neutralization.

### The polyvalent gp120 vaccine induced higher T cell proliferation in immunized hamsters

**Figure 6:**
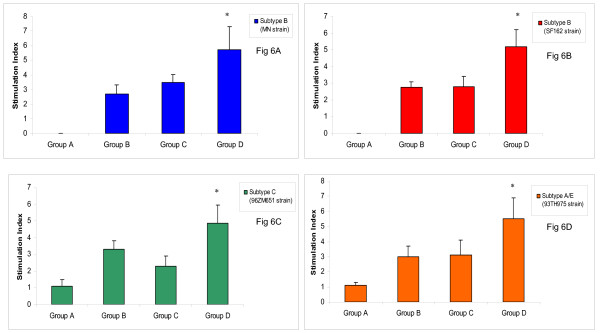
T-cell proliferation results in hamsters immunized with the candidate HIV-1 gp120 vaccines. Splenocytes were cultured and stimulated with 10 μg/ml proteins of (6A): HIV-1 gp120 subtype B (MN strain), (6B): HIV-1 gp120 subtype B (SF162 strain), (6C): HIV-1 gp120 subtype C (96ZM651 strain), and (6D): HIV-1 gp120 subtype A/E (93TH975 strain). Two days later, ^3^H-thymidine was added and after 18 h the incorporated radioactivity was measured in harvested splenocytes. The stimulation index (SI) ± S.E. is shown for each group. Symbol * indicates a significant difference between group D with groups B and C (*p *≤ 0.05).

The proliferation of CD4+T cells plays an important role in both humoral and cellular immune responses by expansion of antigen-stimulated B and CD8+T cells, respectively. A high proliferative immune response was observed to the gp120 protein isolated from patient #001 in all groups with the exception of the control group (data not shown). However, to assess and measure the strength of the candidate vaccines, splenocytes from immunized hamsters were further stimulated with HIV-1 gp120 strains and subtypes that were not included in the vaccine components. Splenocytes were cultured in the presence of HIV-1 gp120 proteins. Two days after stimulation, [^3^H] thymidine was added and sixteen hours later, cells were harvested and thymidine incorporation was measured. The groups B and C showed a relatively similar proliferative immune response. Interestingly, the group of hamsters that was immunized with all 14 gp120 proteins showed the highest lymphocyte proliferative immune response (Fig 6) to subtypes B (MN and SF162), C (96ZM651) and A/E (93TH975), supporting the cross-subtype activity of Th immune responses.

## Discussions and Conclusion

The antigenic variation of HIV-1 gp120 is a major obstacle for the development of an effective vaccine [16-18]. Nabs are mainly directed against the V3 region of gp120 which plays an important function in the binding of virus to either CCR5 or CXCR4 [19]. However, antigenic hypervariability of V1-V5 regions especially V3 assists the virus in escaping neutralization [20-24]. Interestingly, a single mutation in the V3 region, could change R5 virus into an X4 virus [25,26].

It has been shown that mono-or bi-valent HIV-1 envelope vaccines are poorly immunogenic and induce a limited breadth of reactivity. Although Nabs have been shown to control some laboratory-adapted strains, these results cannot easily be extended to primary clinical HIV-1 isolates. In addition, induced Nabs from single HIV-1 envelope studies lacked cross-subtype neutralizing activity and could not neutralize other strains of HIV except the strain contained in the vaccine preparation [27-30]. In an interesting study, Zhan et al., showed that cocktail of 50 HIV-1 envelope in the context of DNA vector, recombinant vaccinia vector and recombinant proteins is able to induce both humoral and cell-mediated immune responses in immunized macaques. Vaccinated macaques showed lower virus titers and higher amount of CD4+T cells compared to unvaccinated animals after challenge with SHIV89.6P. In addition, four out of six vaccinated macaques survived 44 week after the virus challenge, suggesting that a multi-envelope vaccine may be an effective approach to control HIV-1 infection [33].

To increase the breadth of the immune response against HIV-1 strains, ten HIV-1 strains that had a non-syncytium-inducing phenotype and four that had a syncytium-inducing phenotype were selected from a bank of clinical isolates. Although 14 gp120 proteins may not maximize the immune response and may not represent all subtype B isolates; these results my provide clues regarding the capacity of heterologous HIV-1 subtype strains to induce antiviral immune responses that recognize other HIV-1 subtypes. The multivalent approach to increase the breath of the immune response against heterologous strains within a subtype could provide for enhanced vaccine protection within a geographic region.

Sequence analysis from the structure of gp120s revealed that despite vivid antigenic differences between R5 and X4 isolates, the structure of their gp120 core is similar. The sequences of the HIV-1 strains were analyzed by multiple sequences ClustalW alignment program and the phylogenetic tree was constructed using the Neighbor Tree-Maker. The evolutionary history of the cohort gp120 clones was compared to other HIV-1 strains from the same HIV-1 subtype or different subtype. Phylogenetic analysis confirmed that all gp120 clones were derived from subtype B. Gp120 proteins were expressed in the CHO cells to produce a glycosylation pattern similar to the one observed in the native structural form of gp120 found on the virus envelope. Gp120 proteins were poorly expressed in transiently transfected CHO cells. To solve this problem, stable CHO cell lines were created. The production of gp120 proteins were increased more than 20-fold in the stable cell lines, allowing for efficient protein purification.

It may be possible that a cocktail of HIV-1 gp120 proteins containing multiple epitopes will be able to maximize the breadth of immune response against HIV-1 compared to a single immunogen. In a recent study, several macaques were primed by DNA encoding gp120 from subtypes A, B, and C and boosted with recombinant gp120 proteins. Although strong cell-mediated immune responses were induced, a potent neutralizing antibody response was not detected in animals [32]. So far, limited polyvalent vaccine studies have been performed and none have measured immune responses against diverse HIV strains. On the other hand, an effective HIV vaccine may have to deal with not only multiple subtypes but with many strains in each subtype. It is interesting to note that the surface envelope in each HIV subtype has featured differences. For instance, HIV-1 subtype C has enormous genetic differences compared with the recombinant subtypes A/C. The question arises whether HIV-1 immunogens from one subtype will be effective against other subtype. Leandersson et al., showed T-cell reactivity between gp160 immunogen subtype B with other subtypes in HIV individuals [33]. However, they did not detect the neutralizing cross-reactivity between HIV-1 subtypes. Another concern in a polyvalent vaccine approach is that there are no studies regarding the synergistic or antagonistic effects of gp120 genes. It is possible that protein components in a polyvalent vaccine cocktail interact and compete with each other and consequently may inhibit or downgrade the response to some proteins.

Due to high background in mice sera, we were not able to measure the neutralizing antibody responses in our previous vaccine studies, thus, golden hamsters were considered for measurement of the efficacy of humoral immune responses. CD8+T cell immune response was not studied due to non-existent labelled antibodies to hamster CD3 and CD8. In order to analyze the binding antibody titre, plasma from immunized hamsters was collected and HIV-1 gp120 subtypes B (MN, SF162), C (96ZM651) and A/E (93TH975) were analyzed separately by ELISA. Group D, which received all fourteen proteins, showed higher mean IgG antibody titer to HIV-1 subtype B isolates MN and SF162 in comparison to groups B and C. While results from binding assays do not predict neutralization serotypes, they suggest that antigenic subtypes may be related, but not identical, to the genetic subtypes. Theoretically, an augmented number and amount of envelope proteins may enhance the control of viremia following HIV-1 infection through a number of immunologic mechanisms, including increasing the functional activity of B cells, promoted neutralizing antibodies, and cross-reactivity with various HIV-1 subtypes.

In this study, the candidate vaccines were able to neutralize only the HIV-1 strain MN and not any other HIV-1 subtypes. Interestingly, the immunized group with fourteen-gp120 proteins subtype B induced a response similar to the group that received four gp120 proteins. Therefore, it will be useful to evaluate the immunogenicity of each gp120 and interaction with any other gp120 protein in a non-human primate model. These results are similar to a previously published polyvalent study where the neutralization was restricted to the virus strains used for vaccination and the polyvalent vaccine was not able to neutralize any primary HIV-1 isolates [34]. Lack of cross reactivity between Nab results were also consistent to a recent study by Cavacini et al., demonstrating no quantified cross-subtype antibody reactivity and neutralization between subtype B sera against subtype C HIV virus [35]. In another study, Wang et al. showed that a polyvalent HIV-1 envelope protein approach is not able to induce neutralizing antibody responses against HIV-1. However, a polyvalent HIV-1 envelope approach in the context of DNA priming followed by protein boosting may be able to neutralize some of HIV-1 strains from different subtypes [36].

HIV-1 patients are faced with low number of CD4+Th cells in different stages of disease. An inverse correlation between gag-specific Th cells and HIV-1 RNA plasma virus load has been shown. The strongest gag specific proliferative responses were associated with lower viral load, while a high viral load was observed with weak response to gag stimulation [37]. Here, lymphocyte proliferation assay was tested to determine activation of lymphocytes after encountering with HIV-1 gp120 proteins from different subtypes. A relatively high proliferative immune response was determined to subtypes B, C and A/E in the group of hamster that immunized with fourteen gp120 proteins subtype B. This group developed stimulation indices four fold or more above baseline against all subtypes. These results were in consistent with Currier JR et al. that showed a comprehensive cross-subtype reactivity of cell-mediated immune responses between individuals with different HIV-1 subtypes [38]. In summary, the polyvalent gp120 candidate vaccine derived from subtype B was not able to neutralize primary HIV-1 isolates. However, a substantial proportion of cross-subtype CD4 reactivity was observed between the polyvalent group with HIV-1 subtypes B, C, and A/E.

## Methods

### HIV Clinical Isolates

Viruses were isolated from HIV-infected individuals during their visits to the HIV clinic in the Ottawa Hospital. Verbal consent was obtained from HIV patients for participation in the study. Briefly, isolation of virus was performed by placing 2–4 × 10^6 ^peripheral blood mononuclear cells (PBMCs) in Iscove's Modified Dulbecco's Medium (IMDM: Sigma, St. Louis, MO) supplemented with 10% fetal calf serum (FCS: Life Technologies, Grand Island, NY), 2 Mm Glutamine (Sigma) and 50 μg/ml gentamicine (Sigma) with an equal number of phytohemagglutinin (PHA: Sigma) stimulated donor cells. The cells were incubated at 37°C containing 5% CO_2 _and 20 IU/ml Interleukin-2 (IL-2: R&D systems, Minneapolis, MN) was added to increase the growth of these cells. Co-cultures were maintained for up to 4 weeks with periodic media changes. 2 × 10^6 ^freshly stimulated donor cells were added every 10 days to sustain HIV replication. Viruses were measured in the supernatant by HIV p24 antigen kit (DuPont, Wilmington, DE).

### Amplification and cloning of HIV-1 gp120 genes into pEF6-myc-His vectors

Total RNA was purified using RNeasy extraction kit (Qiagen, Mississauga, ON) from syncytium and non-syncytium inducing HIV-1 subtype B co-cultures. The encoding gp120 sequences (1.6 kb) were amplified by RT-PCR. The amplicons were purified using the QIAquick gel extraction kit (Qiagen, Mississauga, ON) and cloned into the PCR 2.1 TOPO-TA vector (Invitrogen, Burlington, ON). After plasmid digestion, the 1.6 kb band corresponding to the gp120 genes were sub-cloned into the pEF6-myc-His vectors (Invitrogen) which is designed for the over-production of recombinant proteins in mammalian cell lines. In this system, recombinant proteins may be purified by affinity chromatography using the c-myc epitope or poly histidine (6 × His) metal-binding tags. The expression constructs were confirmed and characterized by restriction enzymes and nucleotide sequence analysis.

### Characterization of amplified gp120s

All fourteen HIV-1 gp120-V3 regions were aligned using "ClustalW" which is a multiple sequence alignment program for DNA or proteins [39,40]. At the end, the sequences were also manually adjusted. The phylogenetic tree was constructed by the Neighbor Tree-Maker in HIV sequence database. The program converts sequences alignment to the FASTA format.

### Chinese Hamster Ovary (CHO) culture

CHO cells were grown at 37°C, 5% CO_2 _in IMDM (Sigma) supplemented with 10% FCS (Life Technologies), 100 U/ml penicillin and 100 μg/ml gentamicin (Sigma).

### Expression of recombinant proteins

CHO cells were transfected with pEF6-myc-His vectors expressing HIV-1 gp120 structural genes. Stable cell lines were established by using blasticidine-supplemented medium. Cells were harvested, sonicated and lysed in lysis buffer (25 mM Tris base, 2.5 mM Mercaptoethanol, 1% Triton-X100 and a cocktail of protease inhibitors). Recombinant viral proteins were purified with the TALON metal resin kit (Clontech, Palo Alto, CA) as per manufacturer's instructions. The recombinant proteins were confirmed by western blotting and Immunofluorecence antibody staining as previously described [41-43].

### Animal experiments

Six-week old golden hamsters were purchased from Jackson Laboratory (Bar Harbor, ME). Hamsters were maintained in accordance with laboratory standard procedures and were housed in Animal Care in the Faculty of Medicine at University of Ottawa. A total of 100 μg recombinant protein in 0.2 mg of Monophosphoryl lipid A (MPL: Corixa, Hamilton, MT) was injected per animal. Groups of four hamsters were immunized four times subcutaneously (S.C.) in three different sites with one month interval between each immunization. Fourteen days after the last boost, the hamsters were sacrificed and their spleens and blood were collected for further testing or long-term storage in cryopreservation medium.

### Antibody measurement

96-well ELISA plates were coated overnight at 4°C with gp120 proteins clade B (MN, SF162), C (96ZM651) and A/E (93TH975) obtained from the AIDS Research and Reference Reagent Program, National Institute of Allergy and Infectious Diseases (NIAID). The plates were washed with PBS containing 0.05% Tween 20 and then blocked for 1 h with 1% BSA in PBS. Following a second wash with PBS/0.05% Tween 20, the plates were incubated for 2 h at 37°C with serially diluted sera. The plates were washed and incubated for 2 h with peroxidase-conjugated affinity-purified rabbit anti-hamster secondary antibody (Bio-Rad). After washing, color was developed with O-phenylendiamine dihydrochloride (OPD: Sigma, St. Louise, MO). The color reaction was stopped with 1 N HCl and absorbance was read at 490 nm with an ELISA plate reader (Bio-Rad).

### Neutralizing antibody assay

Neutralization was measured as a function of reduction in luciferase reporter gene expression after a single round of infection in TZM-bl cells. TZM-bl cells were obtained from the NIH AIDS Research and Reference Reagent Program, as contributed by John Kappes and Xiaoyun Wu. Briefly, 200 TCID50 of virus was incubated with serial 3-fold dilutions of serum sample in triplicate in a total volume of 150 μl for 1 hr at 37°C in 96-well flat-bottom culture plates. Freshly trypsinized TZM-bl cells (10,000 cells in 100 μl of growth medium containing 75 μg/ml DEAE dextran) were added to each well. Indinavir was added at a final concentration of 1 μM to prevent virus replicaton in the case of HIV-1 MN. One set of control wells received cells + virus (virus control) and another set received cells only (background control). After a 48 hour incubation, 100 μl of cells was transferred to 96-well black solid plates (Corning Costar, Rochester, NY) for measurement of luminescence using Bright Glo substrate solution as described by the supplier (Promega, Madison WI). An assay stock of HIV-1 MN was prepared in H9 cells. Assays stocks of Env-pseudotyped viruses were prepared by tranfection in 293T cells. All virus stocks were made cell-free by 0.45-micron filtration and were stored at -70°C until use.

### Lymphocyte proliferation assay

The assay was performed by [^3^H] thymidine incorporation as previously described [44]. Briefly, splenocytes from immunized hamsters were resuspended at 2 × 10^6 ^cells/ml in RPMI 1640 containing 10% FCS, 50 μM β-mercaptoethanol and 100 U/ml penicillin/streptomycin. A 100 μl aliquot containing 2 × 10^5 ^cells was added to each well of a 96 well plate. The gp120 protein subtypes B, C and A/E (100 μl at 20 μg/ml) were added to each well in triplicate. As a positive control, cells were also stimulated with phorbol 12-myristate 13-acetate and ionomycin (PMA/ION: Sigma). After 48 h of culture, 1 μCi [^3^H] thymidine (Amersham, Arlington Heights, IL) was added to each well. Following 16 h of incubation, cells were harvested onto glass fibre filtermats and thymidine incorporation was measured with a Microbeta beta counter (Wallac, Turku, Finland).

## Abbreviations

Nabs: Neutralizing antibodies

CHO: Chinese hamster ovary

## Authors' contributions

AA performed and analyzed majority of the experiments, participated in the design and wrote the manuscript. DEA participated in analyzing the data. MG helped in the cloning. KG revised the paper critically. FDM participated in the design and coordination of study and helped to draft the final manuscript. All authors read and approved the final manuscript.
